# Introduction of Dengue Virus 4 (DENV-4) Genotype I into Brazil from Asia?

**DOI:** 10.1371/journal.pntd.0000390

**Published:** 2009-04-28

**Authors:** Fernando Lucas de Melo, Camila Malta Romano, Paolo Marinho de Andrade Zanotto

**Affiliations:** LEMB, Department of Microbiology, Institute of Biomedical Science, University of São Paulo, São Paulo, São Paulo, Brazil; University of South Florida, United States of America

Dengue fever is the most important viral neglected tropical disease [1], and during 2008 more than 865,000 cases of dengue infection were reported in the American continent. Brazil is experiencing severe yearly outbreaks of dengue virus (DENV) serotypes 1 to 3 [2]. Since its brief incursion into the eastern Brazilian Amazon in 1982 [3], DENV-4 has not been detected or known to have caused outbreaks in Brazil; as a result, its inclusion in routine epidemiological surveillance protocols was deemed unnecessary by Brazilian health authorities. In 2008, however, DENV-4 was reported in three patients without any history of traveling outside Manaus (Brazil) by academic researchers who did not have any DENV-4 strains in their laboratories [4]. The Brazilian health authorities dismissed the work, not only by not being able to reproduce the findings in their own laboratories, but also by arguing that ultimately there is no apparent DENV-4 outbreak in Brazil.

**Figure 1:**
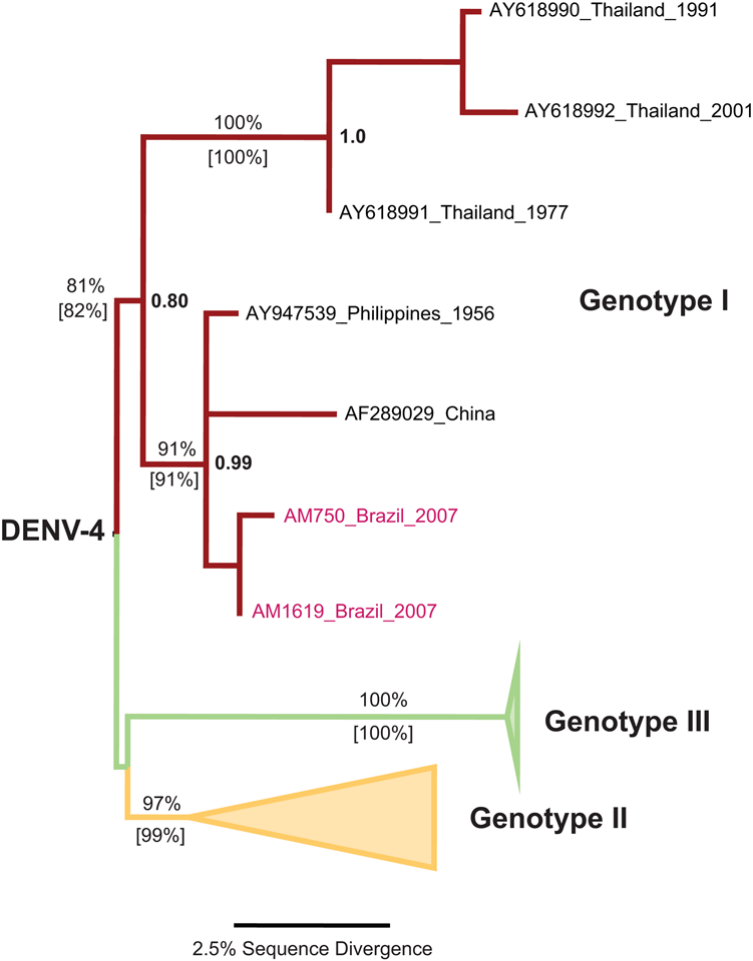
Phylogenetic Tree for DENV-4 Samples from Manaus, Brazil. The tree was inferred with the maximum likelihood criterion implemented in the program GARLI v0.95 [8]. Node support was evaluated with 100 independent runs with GARLI (percent values above branches), 500 non-parametric bootstrap replicates with PhyML [9] (percent values in between brackets below branches), and the posterior probability from the maximum credibility tree among 2,000 trees obtained after running 20 million generations in 15 chains with the parallel implementation of MrBayes v3.0B4 [10] (in bold to the right of the critical nodes). Genotypes II (yellow) and III (green) lineages were collapsed for clarity. The high support values are indicative of the membership in genotype I, but have to be taken cautiously since only 390 bp of the prM-core junction were reported (for more details, see the open-access paper by Figueiredo et al. [4]).

But what could the available sequences from two of the patients actually be showing? To their credit, Figueiredo et al. [4] identified the sequences as DENV-4 using a coarse-grained similarity search with BLAST [5]. Nevertheless, sequences sharing the highest bit-scores in BLAST are not necessarily the closest phylogenetic relatives [6]. Therefore, we have undertaken a more detailed phylogenetic analysis of the controversial samples from Manaus. An initial dataset including reference sequences from all four serotypes was used to classify the samples from Manaus (dataset available upon request). The results supported the previous finding that samples AM750 and AM1619 were indeed DENV-4. A second data set was then assembled containing 34 DENV-4 sequences, including all three DENV-4 genotypes (I, II, and III) known to cause outbreaks around the world [7] (GenBank accession numbers EU127900, EU127899, AY947539, AY618992, AY618991, AY618990, AY618989, AY618988, AY152360, AY152316, AY152312, AY152304, AY152300, AY152292, AY152260, AY152252, AY152212, AY152148, AY152144, AY152132, AY152112, AY152108, AY152104, AY152100, AY152096, AY152092, AY152088, AY152084, AY152076, AY152064, AY152060, AY152052, AF326573, and AF289029). Crucially, both samples from Manaus were sister taxa and nested within Asian genotype I (Figure 1, see legend for methods used) using a genetic algorithm–based method [8]. This result was also obtained more than 91% of the time during maximum likelihood non-parametric bootstrap with the PhyML program [9] (Figure 1). Moreover, it also had a high Bayesian posterior probability (0.99) while using a Markov chain Monte Carlo inference-based method in the MrBayes program [10] (Figure 1). Remarkably, the samples isolated in Manaus in 2007 were distinct, which would be expected from viruses isolated from different individuals in a web of transmission. We further substantiated our findings by comparing the likelihood of the observed tree (*lnL* = −1588.53) to those estimated for alternative groupings into genotype II (*lnL* = −1705.85) and III (*lnL* = −1702.88). We argue that the likelihood differences in combination with high non-parametric bootstrap values and high posterior probabilities (Figure 1) constitute considerable evidence that DENV-4 genotype I strains may be in fact circulating in Brazil. Nevertheless, even with the high levels of support obtained during our analysis, we would argue that additional sequence information is necessary to further describe these strains.

While genotype II has been present in the Americas for over two decades, the presence of DENV-4 genotype I in Brazil was a rather unexpected result, since genotype I is not known to be circulating in the West [3],[11],[12]. Moreover, we would expect its emergence in Central America and the Caribbean first, following the pattern of introduction of many other DENVs [13], but the intensification of economic activities between Brazil and other Asian countries (especially China) could explain a direct introduction to Brazil that bypasses the Caribbean. These are preliminary, albeit potentially troublesome, results, and it begs checking if these strains entered Brazil directly or if DENV-4 genotype I is already experiencing cryptic circulation elsewhere in the Americas. Although DENV-4 is less prevalent (2%), it has been shown to be involved in 10% of dengue hemorrhagic fever cases following secondary infections in children in Thailand [14]. In sum, the presence of DENV-4 in the Americas should be taken with great caution and warrant an intensification of the ongoing surveillance activities in Brazil in particular, where routine serology and nucleic acid detection methodology that includes DENV-4 should be considered.
